# Extractions of High Quality RNA from the Seeds of Jerusalem Artichoke and Other Plant Species with High Levels of Starch and Lipid

**DOI:** 10.3390/plants2020302

**Published:** 2013-04-29

**Authors:** Tanupat Mornkham, Preeya Puangsomlee Wangsomnuk, Yong-Bi Fu, Pinich Wangsomnuk, Sanun Jogloy, Aran Patanothai

**Affiliations:** 1 Department of Biology, Faculty of Science, Khon Kaen University, Khon Kaen 40002, Thailand; E-Mails: mtanupat@yahoo.com (T.M.); pinwan@kku.ac.th (P.W.); 2 Plant Gene Resources of Canada, Saskatoon Research Centre, Agriculture and Agri-Food Canada, Saskatoon, SK S7N 0X2, Canada; E-Mail: yong-bi.fu@agr.gc.ca; 3 Department of Plant Science and Agricultural Resources, Faculty of Agriculture, Khon Kaen University, Khon Kaen 40002, Thailand; E-Mails: sanun@kku.ac.th (S.J.); aran@kku.ac.th (A.P.)

**Keywords:** RNA extraction, polysaccharide, lipid, Jerusalem artichoke, TRIzol^®^

## Abstract

Jerusalem artichoke (*Helianthus tuberosus* L.) is an important tuber crop. However, Jerusalem artichoke seeds contain high levels of starch and lipid, making the extraction of high-quality RNA extremely difficult and the gene expression analysis challenging. This study was aimed to improve existing methods for extracting total RNA from Jerusalem artichoke dry seeds and to assess the applicability of the improved method in other plant species. Five RNA extraction methods were evaluated on Jerusalem artichoke seeds and two were modified. One modified method with the significant improvement was applied to assay seeds of diverse Jerusalem artichoke accessions, sunflower, rice, maize, peanut and marigold. The effectiveness of the improved method to extract total RNA from seeds was assessed using qPCR analysis of four selected genes. The improved method of Ma and Yang (2011) yielded a maximum RNA solubility and removed most interfering substances. The improved protocol generated 29 to 41 µg RNA/30 mg fresh weight. An A260/A280 ratio of 1.79 to 2.22 showed their RNA purity. Extracted RNA was effective for downstream applications such as first-stranded cDNA synthesis, cDNA cloning and qPCR. The improved method was also effective to extract total RNA from seeds of sunflower, rice, maize and peanut that are rich in polyphenols, lipids and polysaccharides.

## 1. Introduction

Jerusalem artichoke (*Helianthus tuberosus* L.) is a member of family Asteraceae, native to North America, and a wild relative of the cultivated sunflower (*H. annuus* L.). This species is an old tuber crop and recently has received renewed interest in terms of genetic improvement. The Jerusalem artichoke plant can be grown for human consumption, alcohol and fructose syrup production, and livestock feed. Jerusalem artichoke tubers accumulate inulin as a carbon source that can be used as raw material to produce a variety of value-added products [1]. Thus, the efforts to develop improved Jerusalem artichoke cultivars have increased over the last decades. However, little attention has been paid to Jerusalem artichoke seed development, and the molecular mechanism of seed germination remains poorly understood. Clearly, molecular analysis of seed development is warranted to enhance the efficiency of seedling selection leading to success in the development of Jerusalem artichoke cultivars with desirable traits.

Plants reveal a huge morphological and physiological diversity in seed types and states to match local environmental demands for germination timing [2]. Such diversity imposes more challenges to understand the gene expressions involved in seed dormancy and germination, the important stages of the seed plant life cycle. It is possible to perform molecular analysis of these physiological mechanisms through PCR, RT-PCR (reverse transcriptase-PCR) and also quantitative real-time PCR (qPCR), but these PCR-based methods require high-quality DNA-free RNA. Several seed-RNA extraction protocols have been developed for *Arabidopsis*, rice, wheat, maize, sorghum, sunflower, peanut, and olive [3,4,5,6,7,8,9], but no applications of these methods have been found to extract total RNA from Jerusalem artichoke seeds.

The basic idea behind the RNA extraction is relatively simple, but it is difficult to deliver reliable quantity and purity of RNAs for molecular analyses of an under-exploited tissue such as Jerusalem artichoke seeds, which contains high levels of polysaccharides and lipid. The Jerusalem artichoke seeds were reported to have 45.5% oil [10]. Seed yield per Jerusalem artichoke plant varies with genotype, location and production conditions, as Jerusalem artichoke flowers are often sterile [11,12]. These features, along with low germination and dormancy of Jerusalem artichoke seeds, make the extraction of total RNA more challenging [6,13].

The objectives of this study were to improve existing methods for extracting total RNA from Jerusalem artichoke seeds and to assess the applicability of the improved method in other plant species. Specifically, this study assessed five existing RNA extraction methods and modified the methods of Li and Trick [3] and Ma and Yang [6] (or MLT and MMY methods for short, respectively). The MMY protocol was further applied to assay seeds of diverse Jerusalem artichoke accessions, sunflower, rice (*Oryza sativa* L.), maize (*Zea mays* L.), peanut (*Arachis hypogaea* L.) and marigold (*Tagetes erecta* L.) and the extracted RNAs were subjected to downstream applications.

## 2. Results and Discussion

**Figure 1:**
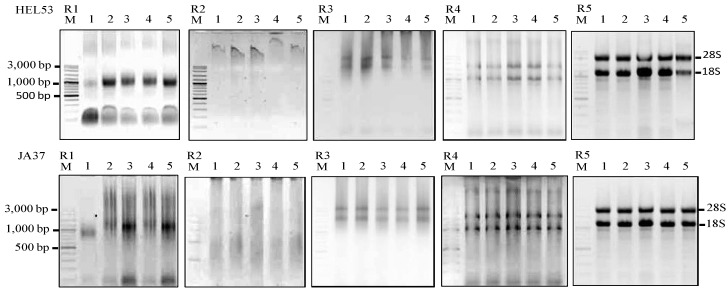
Agarose gels showing the extractions of total RNA using five RNA isolation methods (R1–R5) from five dry seeds of two Jerusalem artichoke accessions (HEL53, the top panel; JA37, the bottom panel). The five isolation methods are the TRIzol^®^ (Invitrogen), R1; Plant RNeasy mini kit (Qaigen), R2; Method of Wang *et al.* [7], R3; MLT method, R4; and MMY method, R5. M is 100 bp DNA ladder plus (Vivantis). The five dry seed samples are numerically labeled from 1 to 5.

Our initial attempt using the five existing methods to extract total RNA from Jerusalem artichoke dry seeds yielded variable, unsatisfactory results (Table 1 and Figure 1). The resulting yields of total RNA were unexpectedly low, smaller than 15 µg RNA/30 mg fresh weight. Clearly, the efforts with the plant RNeasy^®^ method (Qaigen, Hilden, Germany) and TRIzol^®^ reagent (Invitrogen, Carlsbad, CA., USA) were not successful. The pellet formed a viscous substance, and RNA recovery was very poor, even though these commercial kits have been successfully used in our lab to extract RNA from leaf, root, shoot, flower and tuber of Jerusalem artichoke [14].

**Table 1:** Average yield and purity of total RNA using each of five RNA extraction.

RNA Method	Absorption ratio		Absorption ratio		RNA concentration	
	(260 nm/280 nm) ^#^		(260 nm/230 nm) ^#^		(µg/30 mg fresh weight) ^#^	
	JA37	HEL53	JA37	HEL53	JA37	HEL53
1. TRIzol	1.76 ^c,d^	1.74 ^b^	0.69 ^c^	0.68 ^c^	5.26 ^d^	5.31 ^d^
2. Plant RNeasy mini kit (Qaigen)	1.74 ^d^	1.67 ^c^	0.54 ^c^	0.54 ^c^	1.04 ^e^	1.00 ^e^
3. Wang *et al.* method [7]	1.82 ^b,c^	1.77 ^b^	1.56 ^b^	1.55 ^b^	9.16 ^c^	12.84 ^c^
4. Li and Trick method [3]	1.80 ^c,d,e^	1.81 ^c,d,e^	1.26 ^b^	1.29 ^b^	6.69 ^d^	6.67 ^d^
5. Ma and Yang method [6]	2.15 ^a^	2.16 ^a^	1.05 ^c^	1.06 ^c^	1.04 ^f^	1.10 ^f^
6. MLT method	1.90 ^a^	1.86 ^a^	2.24 ^a^	1.87 ^a^	15.53 ^b^	15.24 ^b^
7. MMY method	1.84 ^b^	1.83 ^a^	1.74 ^b^	1.79 ^a^	34.67 ^a^	34.56 ^a^
LSD	0.04 *	0.04 *	0.07 *	0.07 *	0.32 *	0.35 *

Methods from five seed samples of two Jerusalem artichoke accessions (JA37 and HEL53). ^#^ Values with different superior letters (a–f) within column are significantly different at *p* < 0.05 level by LSD. * Significant at *p* < 0.05 level.

Commercial RNA isolation kits were not designed for use with plant tissues containing high concentrations of polyphenols, polysaccharides and other secondary metabolites. There are reports modifying extraction buffers in the plant RNeasy^®^ mini kit and leading to successful RNA extraction on leaves and bud tissue of grapevine and from leaves, bud and cane tissues of woody plant [15,16], but this has rarely been tested on seeds. Applying the published protocols developed for RNA isolation from cereal seeds [3,7] and sunflower dry seeds [6] generated poor quality and low yield RNA from Jerusalem artichoke dry seeds. We also observed that the pellet became viscous when phenol and chloroform were added. The method of Wang *et al*. [7] was able to extract 9 and 13 µg RNA/30 mg fresh weight from Jerusalem artichoke cv. JA37 and HEL53, respectively. A260/A280 were 1.82 and 1.77 while A260/A230 ratios of total RNA were 1.56 and 1.55 (Table 1), suggesting their impurity. 

Considering the high levels of starch and lipid present in Jerusalem artichoke seeds, we modified the methods of Li and Trick [3] and Ma and Yang [6] by adjusting the amounts of reagents and adding extra important steps. Specifically, the original Li and Trick [3] method was modified as MLT method by adding a step containing TRIzol^®^ reagent which contains phenol and guanidine isothiocyanate, a strong denaturant. This solution helps to remove all traces of interfering protein and other metabolites [17]. The addition of 750 µL instead of 400 µL of extraction buffer and using 500 µL of TRIzol^®^ reagent instead of 250 µL of extraction buffer II in Li and Trick [3] protocol helped to get 16 and 15 µg RNA/30 mg fresh weight from seeds of Jerusalem artichoke cv. JA37 and HEL53 within approximately one and a half hours (Table 1; Figure 1) which is higher than the amount of RNA extracted from more seed weight (60 mg) using the original protocol of Li and Trick [3] (Table 1). These efforts generated successful extractions of total RNA from Jerusalem artichoke dry seeds, as illustrated in Table 1 and Figure 1. The yields of total RNA by MLT were significantly higher than those obtained using the other unmodified methods.

The original method of Ma and Yang [6] utilized high salt concentration (8 M LiCl) and β-mercaptoethanol in the extraction buffer to inactivate RNase activity. The extraction and solubilization buffers, in the presence of PVP and SDS in two-steps RNA isolation of Ma and Yang [6] helped to remove polysaccharides, proteins and polyphenolic compounds [17]. PVP also prevented the oxidation of polyphenols, which occur after long air exposure of samples. The polyphenol removal was carried out using two repeat chloroform extractions prior to RNA extraction using Trizol^®^. We improved the original protocol further as MMY method with several considerations. These included (1) the reduction of seed sample to 30 mg; (2) the change in the temperature and incubation time to room temperature for 5 min; (3) purification of a pellet instead of the suspension in the original protocol of Ma and Yang [6] after adding chloroform to the mixture of fine powder, the extraction buffer and β-mercaptoethanol, and ethanol; LiCl precipitated RNA as a pellet, leaving DNA in the supernanant; (4) the replacement of NaAc-saturated acidic phenol and chloroform by Trizol^®^ reagent and chloroform; and (5) precipitation of total RNA using isopropanol instead of ethanol and 3 M NaAc, pH 5.2. These modifications helped to overcome the problem of viscous pellets in the original method of Trizol^®^ and produced significant higher yields of total RNA (35 µg RNA/30 mg) than those using the original method of Ma and Yang [6] and also the MLT method (Table 1; Figure 1). The MMY method also made the RNA extraction effort simpler and more reproducible and the overall effort required approximately one and a half hours. The quality of RNA prepared by this method was demonstrated by intact sharp 28S and 18S rRNA bands and no sign of RNA degradation on agarose gel. The extracted RNA was of high quality indicated by the A260/A280 and the A260/A230 ratios (Figure 1 and Table 1) [18,19]. 

**Figure 2:**
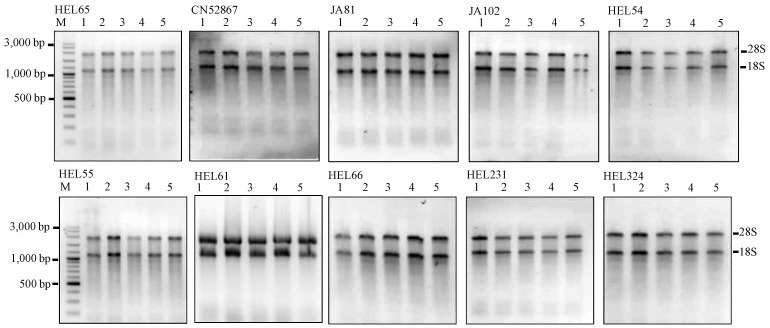
Agarose gels showing the extractions of total RNA using the MMY method from five dry seeds of 10 additional Jerusalem artichoke accessions. Each sub-panel represents the extractions of total RNA labeled for each accession. M is 100 bp DNA ladder plus (Vivantis). The five dry seed samples are numerically labeled from 1 to 5.

To assess the efficiency of the MMY method, we further assayed another set of dry seeds from 10 extra JA accessions of diverse origins (listed in Table 2). The results are encouraging and proved to be applicable for all Jerusalem artichoke seeds independent of their mother plants (Figure 2 and Table 3). 

**Table 2:** Eighteen assayed samples of Jerusalem artichoke and other plant species.

Sample	Origin/Source
*Jerusalem artichoke* ^a^	
CN52867	The former Union of Soviet Socialist Republics (USSR)
JA37	Canada
JA81	France
JA102	Germany
HEL53	Germany
HEL54	Germany
HEL55	Germany
HEL61	The former Union of Soviet Socialist Republics (USSR)
HEL65	The former Union of Soviet Socialist Republics (USSR)
HEL66	Ukraine
HEL231	Germany
HEL324	Unknown
*Other plant species*	
Sunflower cv. Beauty Mix	Chia Tai Co. Ltd., Thailand
Maize cv. Tien Khao (pale colour)	Khon Kaen University, Thailand
Maize cv. Tien Dam (dark purple)	Khon Kaen University, Thailand
Rice cv. KDML105	Khon Kaen University, Thailand
Peanut cv. 4401	Khon Kaen University, Thailand
Marigold	Chua Yong Seng Seed Co. Ltd., Thailand

^a^ see Kays and Nottingham [12] for Jerusalem artichoke sample description.

**Table 3:** Average yield and purity of total RNA extracted using the MMY method in five seed samples of each accession representing six plant species.

Plant species	Absorption ratio	Absorption ratio	RNA Concentration
	(260 nm/280 nm)^#^	(260 nm/230 nm) ^#^	(µg/30 mg fresh weight) ^#^
Jerusalem artichoke CN52867	1.80 ^c,d^	2.21 ^c,d,e^	33.30 ^b,c,d,e,f^
Jerusalem artichoke JA37	1.84 ^c,d^	1.74 ^f,g^	34.67 ^b,c,d,e^
Jerusalem artichoke JA81	1.90 ^b,c^	2.33 ^c,d^	33.23 ^b,c,d,e,f^
Jerusalem artichoke JA102	1.82 ^c,d^	2.31 ^c,d,e^	34.36 ^b,c,d,e^
Jerusalem artichoke HEL53	1.83 ^c,d^	1.79 ^f,g^	34.56 ^b,c,d,e^
Jerusalem artichoke HEL54	2.20 ^a^	3.00 ^a^	31.79 ^d,e,f^
Jerusalem artichoke HEL55	2.22 ^a^	3.11 ^a^	29.55 ^e,f^
Jerusalem artichoke HEL61	1.86 ^c,d^	2.52 ^b^	41.01 ^b,c^
Jerusalem artichoke HEL65	1.79 ^c,d^	2.13 ^e^	32.09 ^d,e,f^
Jerusalem artichoke HEL66	1.80 ^c,d^	2.16 ^d,e^	31.16 ^d,e,f^
Jerusalem artichoke HEL231	1.85 ^c,d^	2.22 ^c,d,e^	29.32 ^e,f^
Jerusalem artichoke HEL324	1.80 ^c,d^	2.18 ^c,d,e^	32.58 ^d,e,f^
Sunflower cv. Beauty Mix	1.62 ^e^	1.62 ^g,h^	56.66 ^a^
Maize cv. Tien Khao	1.98 ^b^	2.34 ^c^	41.32 ^b^
Maize cv. Tien Dam	1.90 ^b,c^	2.13 ^e^	25.43 ^f^
Jasmine rice cv. KDML105	1.75 ^d^	1.46 ^h,i^	32.77 ^c,d,e,f^
Peanut cv. 4401	1.85 ^c,d^	1.85 ^f^	62.93 ^a^
Marigold	1.60 ^e^	1.36 ^i^	38.06 ^b,c,d^
LSD	0.11 *	0.18 *	8.35 *

# Values with different superior letters (a–f) within a column are significantly different at *p* < 0.05 level by LSD. * Significant at *p* < 0.05 level.

The yields ranged from 29 to 41 µg RNA/30 mg fresh weight. These results helped to demonstrate the advantage of the improved method in rapid isolation of RNA from small amounts compared to other seed RNA extraction protocols [3,6,7,20,21,22,23]; the latter mostly used 50–100 mg seed material. 

**Figure 3:**
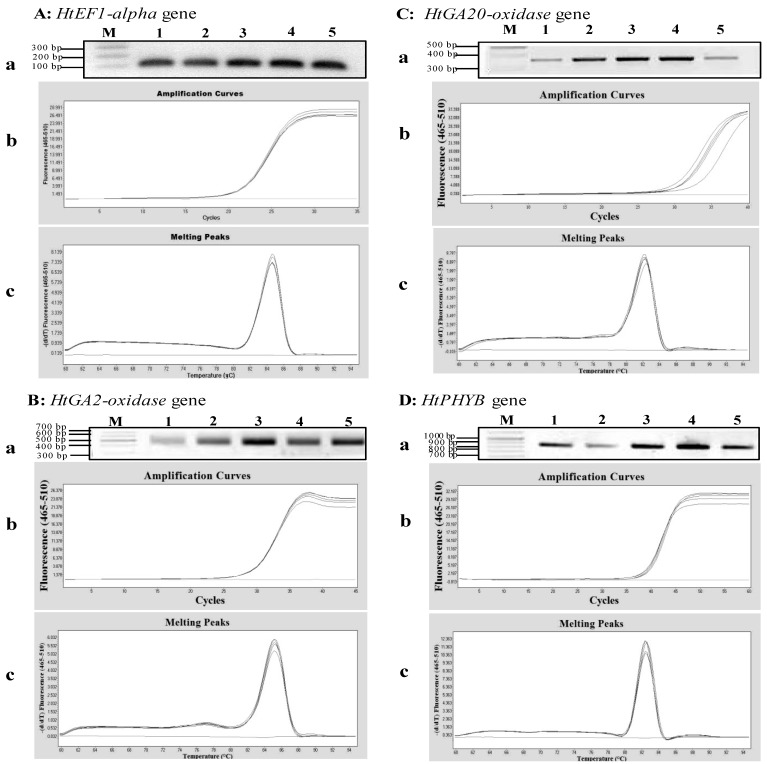
qPCR analyses of four genes expressed in Jerusalem artichoke HEL53 dry seeds. (**A**) the amplification of an 105 bp fragment of the *HtEF1-alpha* gene. (**B**) the amplification of a 535 bp fragment of the *HtGA2-oxidase* gene. (**C**) the amplification of a 373 bp fragment of the *HtGA20-oxidase* gene. (**D**) the amplification of an 845 bp fragment of the *HtPHYB* gene. In each sub-panel for each gene, (**a**) the RT-PCR amplification of genes based on the templates obtained from 1st-stranded cDNA from five dry seeds, on which M is 100 bp plus DNA ladder (Vivantis); (**b**) amplification curves of the PCR products for each gene expressed in the dry seeds; and (**c**) melting peaks of the PCR products for amplified gene cDNA.

To evaluate the integrity of the extracted RNA using the MMY method, qPCRs were used to amplify a housekeeping gene (*HtEF1-alpha*) and genes (*HtGA2-oxidase*, *HtGA20-oxidase* and *HtPHYB*), which are known to play specific roles in the induction of seed germination in *Arabidopsis* [24,25]. The synthesis of cDNAs using the extracted RNA and reverse transcriptase enzyme was successful. The target bands with expected sizes of the *HtEF1-alpha*, *HtGA2-oxidase*, *HtGA20-oxidase* and *HtPHYB* genes were observed (Figure 3). The qPCR cycle thresholds (Ct) was between 18 and 40 cycles, and the melting curve was specific, with a single peak occurring at about 84 °C, 82 °C, 82 °C and 82 °C for *HtEF1-alpha*, *HtGA2-oxidase*, *HtGA20-oxidase* and *HtPHYB*, respectively (Figure 3). These results indicated that the MMY method was effective in extracting total RNA for the transcript analysis of gene expression in Jerusalem artichoke seeds.

**Figure 4:**
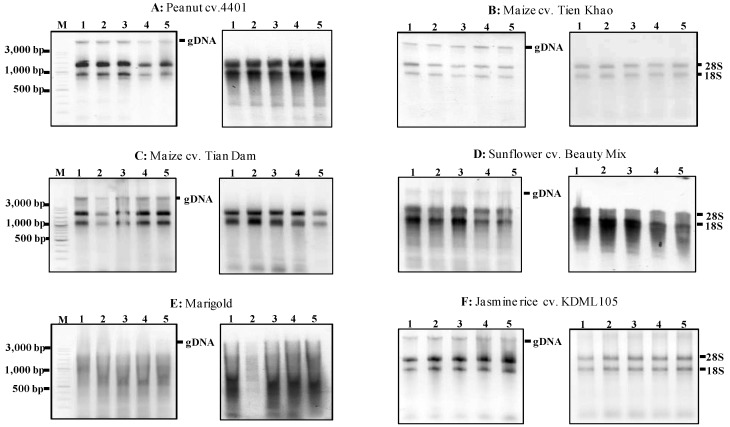
Agarose gels showing the extractions of total RNA using the MMY method from five dry seeds of five other plant species. Each of the six sub-panels for plant species or varieties consists of two gels showing the extractions of total RNA without and with the treatment of DNase (on the left and right, respectively). M is 100 bp DNA ladder plus (Vivantis). The five dry seed samples are numerically labeled from 1 to 5.

We also assessed the applicability of the MMY method to extract total RNA from other plant species with high levels of starch and lipid. Specifically, we isolated total RNA from the seeds of the monocotyledonous crop species maize and rice, from sunflower and marigold as well as from the dicotyledonous crop species peanut (Table 2). The initial assessment revealed that genomic DNA was still contaminated in all plant species studied except Jerusalem artichoke. Thus, the genomic DNA was removed by DNaseI digestion following phenol/chloroform purification. The RNA was then precipitated. Figure 4 illustrates the absence of undesirable contaminants. The yield of total RNA isolated from these seeds ranged from 25–63 μg/30 mg fresh weight (Table 3). 

RNA absorbance (A260/280 and A260/230) values of sunflower and marigold are below 1.8 showing the impurity of protein (Table 3). The Ma and Yang [6] original protocol revealed low purity (A260/280 was 1.50) and low yield of total RNA (1.71 μg/100 mg) from dry sunflower seeds when 350 µL of ethanol was added to the extraction buffer. The total RNA extracted here is much higher than that of Ma and Yang [6] original protocol. As illustrated in Figure 4, the MMY method produced large amounts of total RNA from rice, maize and peanut seeds (Figure 4 and Table 3).

**Figure 5:**
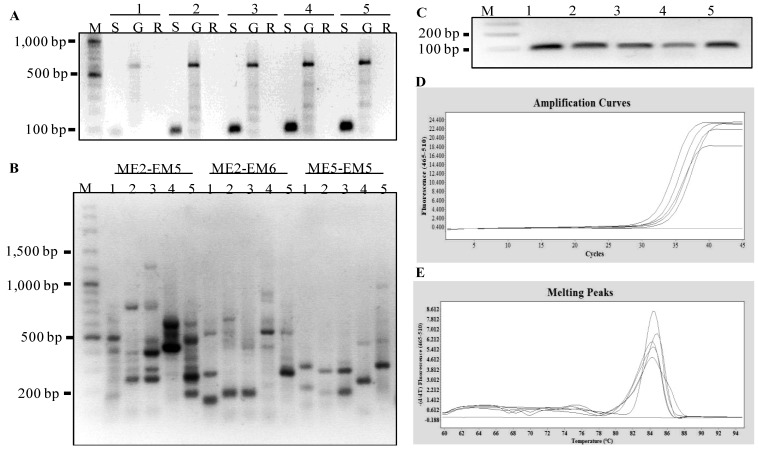
Molecular analyses of gene expressed in five dry seed samples representing four other plant species. The five seed samples are numerically labeled as 1: peanut cv. 4401, 2: maize cv. Tien Khao, 3: maize cv. Tien Dam, 4: sunflower cv. Beauty Mix, and 5: Jasmine rice cv. KDML105. (**A**) PCR amplification of *EF1-alpha* gene based on the templates obtained from 1st-stranded cDNA, genomic DNA and total RNA; these three templates are labeled with the letters S, G and R, respectively. (**B**) the agarose gel showing the SRAP-cDNA amplifications using three primer combinations following Mornkham *et al*. [26] from 1st-stranded cDNAs produced from extracted seed RNAs. (**C**) qPCR amplification of a 105 bp fragment of the *EF1-alpha* gene for five seed samples using HtEF1_F and HtEF1_R primers. (**D**) amplification curves of the PCR products for *EF1-alpha* gene. (**E**) melting peaks of the PCR products for *EF1-alpha* gene.

To further evaluate the integrity of the total RNA extracted from these assayed species, SRAP-cDNA amplifications and a transcript analysis of *EF1-alpha* gene was performed. Figure 5 showed the gene expression was successfully detected in the seeds of peanut, maize, sunflower, and rice. However, the RNAs extracted from marigold were not adequate in these downstream analyses. Clearly, the improved method was effective in extracting RNA of desired quality and quantity from small amount of seeds from several other plant species studied here.

## 3. Experimental Section

### 3.1. Plant Material

This study assayed 18 seed samples of Jerusalem artichoke, sunflower, maize, rice, peanut and marigold (Table 2). Achenes of 12 Jerusalem artichoke genotypes were harvested in 2011 from plants growing in the farm field at Khon Kaen University and JA germplasm was kindly donated by the Plant Genetic Resources of Canada, Saskatoon Research Centre, and by the Leibniz Institute of Plant Genetics and Crop Plant Research (IPK), Germany. The harvested achenes were air-dried and stored at room temperature, and kept at 4 °C until use. The exocarps were removed to obtain seeds just before the total RNA extraction. The seed samples of peanut and maize were obtained from related breeding programs at Khon Kaen University. The seed samples of sunflower and marigold were acquired from Chia Tai Co. Ltd. and Chua Yong Seng Seed Co. Ltd., respectively. The Jasmine rice cultivar was obtained from Khon Kaen Rice Research Center.

### 3.2. RNA Extraction

Total RNA was initially extracted from 30 mg ground seed material with five independent replications using five existing extraction methods. The methods are (1) TRIzol^®^ reagent (Invitrogen), (2) Plant RNeasy mini kit (Qaigen, Germany), (3) method of Wang *et al*. [7], (4) method of Li and Trick [3], and (5) method of Ma and Yang [6]. These initial efforts generated an unsatisfactory amount and purity of total RNA, and further efforts were made to improve the methods of Li and Trick [3] and Ma and Yang [6]. Before operation, all plastic materials were autoclaved. Glass material and the mortar and pestle were baked for 3–6 h at 180 °C.

### 3.3. Solution and Reagents

#### 3.3.1. RNA Extraction Buffer

1a. For the MLT method below, 100 mM Tris-HCl (pH 8.0), 150 mM LiCl, 50 mM EDTA, 1.5% Sodiam Dodecyl Sulfate, add 11.25 µL of β-mercaptoethanol just before use.

1b. For the MMY method below, 8 M LiCl, 2% (w/v) PVP40, and 5% (v/v) β-mercaptoethanol (just before use).

#### 3.3.2. Solubilization Buffer

1.4% (w/v) SDS, 0.075 M NaCl, 0.025 M EDTA, add 2% (v/v) β-mercaptoethanol just before use.

#### 3.3.3. Other Reagents

TRIzol^®^ reagent (Invitrogen), Phenol Solution (Research Organics Inc., Cleveland, OH, USA, saturated with 0.1 M citrate buffer pH 8.0), DNase I (USB Corporation), ethanol, chloroform, isoamyl alcohol and isopropanol of analytic purity.

### 3.4. MLT Method

(1) Thirty mg seeds were ground with mortar and pestle in the presence of liquid nitrogen. Transfer immediately into 1.5 mL microcentrifuge tubes.

(2) A 750-µL extraction buffer was added and mixed by vortex.

(3) The high molecular weight impurities and lipids were removed by adding 750 µL of phenol: chloroform (1:1), mixed gently and centrifuged at 12,000 rpm for 10 min.

(4) The supernatant was collected and the phenol-chloroform extraction was repeated. The supernatant was transferred to a new RNase-free tube.

(5) Supernatant was added with an equal volume (about 500 µL) of TRIzol^®^ reagent, mixed thoroughly and incubated at room temperature for 10 min.

(6) An equal volume of chloroform: isoamyl alcohol (24:1) was added to the tube, mixed gently and centrifuged at 12,000 rpm for 10 min.

(7) The supernatant was transferred to a new RNase-free tube. One volume of isopropanol (about 500 µL) and 200 µL of 1.2 M NaCl were added and mixed by inversion several times, and incubated at −20°C for 15 min. The pellet was precipitated by centrifugation at 10,000 rpm for 10 min. The supernatants were discarded and the RNA pellets were washed carefully with 70% ethanol.

(8) The RNA pellets were re-suspended in 30-µL RNase-free water and stored at −70 °C.

The modified protocol MLT requires a lower amount of seed (30 mg *vs*. 50–100 mg), more extraction buffer (750-µL *vs*. 400-µL), a repeat of the phenol-chloroform extraction (750-µL *vs*. 250-µL) and replace 70% guanidinium sulfate (w/v), 0.75 M sodium citrate, 10% laurylsarcosine, 2 M sodium acetate, pH 4.0 with TRIzol^®^ reagent.

### 3.5. MMY Method

(1) Thirty mg seed was ground with mortar and pestle in the presence of liquid nitrogen and PVP40. 

(2) Transfer immediately into 1.5 mL centrifuge tubes. Then, 0.9 mL of the extraction buffer and 350 µL of ethanol were added, mixed by vortex, and incubated at room temperature for 5 min. 

(3) The high molecular weight impurities and lipids were removed by adding 100 µL chloroform mixed gently and centrifuged at 5,000 rpm for 3 min. 

(4) The supernatant was discarded. The pellet was dissolved in 550 µL solubilization buffer. The chloroform extraction was repeated with equal volume of solubilization buffer and centrifuged at 5,000 rpm for 3 min. 

(5) The supernatant was transferred to a new RNase-free tube and added with 500 µL of TRIzol^®^ reagent and 100 µL of chloroform, mixed thoroughly, incubated at room temperature for 2 min and centrifuged at 12,000 rpm for 10 min. 

(6) The aqueous phase was transferred to a new RNase free tube. An equal volume of isopropanol was added and incubated at −20 °C for 15 min. The pellet was precipitated by centrifugation at 12,000 rpm for 10 min. The supernatant was discarded and the pellet was allowed to dry, followed by re-suspending the pellet in 30 µL RNase-free water. 

The MMY protocol requires a lower amount of seed (30 mg *vs*. 60 mg), more ethanol (350-µL *vs*. 250-µL) and a much shorter incubation time after adding extraction buffer (5 min at room temperature instead of overnight at 4 °C). The purification of pellet was made instead of the suspension in the original protocol of Ma and Yang [6] after adding chloroform to the mixture of fine powder, the extraction buffer and β-mercaptoethanol. Also, the supernatant was added with 500 µL of TRIzol^®^ reagent and 100 µL of chloroform instead of using 500 µL NaAc-saturated acidic phenol and 300 µL chloroform in the second round of purification. 

### 3.6. RNA Extraction from Seeds of Other Plant Species

RNA extraction from seeds of other plant species was done according to the MMY method, as described above. After the RNA precipitation with isopropanol, the pellet was dissolved in 26 µL of RNase-free water and added with 3 µL buffer 10× buffer and 1 µL (10 units) of RQ1 RNase-Free DNase (Promega). Incubate 30 min at 37 °C, followed by adding the RNase-free water (470 µL). The phenol: chloroform (1:1) extraction was repeated with equal volumes (about 500 µL) and centrifuged at 12,000 rpm for 10 min. The supernatant was transferred to a new RNase-free tube, followed by adding an equal volume of isopropanol and 200 µL of 1.2 M NaCl. Incubate at −20 °C for 15 min. The pellet was precipitated by centrifugation at 12,000 rpm for 15 min. The supernatant was discarded and the pellet was allowed to dry followed by re-suspended the pellet in 30 µL RNase-free water. 

### 3.7. RNA Evaluation

The concentration and quality of total RNA extracted from all five existing methods were analyzed based on the 260 nm/280 nm and 260/230 absorbance ratios. An aliquot of total RNA was used in the Agilent 8453 UV-visible spectrophotometer according to manufacturer’s instructions. The yield of total RNA extracted from 30 mg fresh weight was reported. The variation in the efficiency of RNA extractions was analyzed using Statistix 8 [27]. RNA integrity was evaluated from the 28S and 18S rRNA bands from 5 µL of total RNA on 1.0% agarose gel electrophoresis. The gels were stained with ethidium bromide and visualized with UV light. The photographs were taken using Vilber Lourmat (France) and the images were inverted in Adobe Photoshop. The evaluations were initially performed on the total RNA using the five extraction methods, followed by those using the MMY method from the 18 seed samples representing Jerusalem artichoke, sunflower, maize, rice, peanut and marigold. 

### 3.8. Reverse Transcription and Subsequent Application

Transcript expression of selected genes was quantified by qPCR. After DNase treatment using RQ1 RNase-Free DNase (Promega), five replicates of 1.25 µg of total RNA from Jerusalem artichoke seeds were reverse-transcribed in a 10 µL reaction using OligodT (2.5 µM) and digested with RNaseH according to the SuperScript™ Vilo kit instructions (Invitrogen). After the first strand cDNA synthesis, the solution was 10× diluted to a final concentration of 125 ng/µL. PCR amplification was made based on 2 µL of diluted 1st strand-cDNA using *Elongation factor 1-alpha* (*HtEF1-alpha*), *HtGA2-oxidase*, *HtGA20-oxidase*, and *Phytochrome B* (*HtPHYB*) gene-specific oligonucleotide primers (Table 4). These primers were designed based on gene homologies from the Compositae Genome Project Database [28] which were confirmed using BLAST. The qPCR experiments were performed in a thermocycler called “LightCycler^®^ 480” (Roche). The reaction was conducted with a final volume of 20 µL using the LightCycler^®^ 480 SYBR Green I Master kit (Roche) according to the manufacturer’s instructions. The qPCR condition was as follows: at 94 °C for 5 min; 45 cycles of 94 °C for 30 s, 55 °C for 30 s and 72 °C for 30 min, with a final extension at 72 °C for 5 min. The PCR products were also analyzed on 1.0% agarose electrophoresis and visualized as described above. The gene expression analysis was done based on the total RNA extracted from seeds of Jerusalem artichoke and other assayed plant species.

**Table 4:** Primer sequences used for gene assay in this study.

Gene ID/primer	Primer name	Sequence	Calculated Tm (°C) ^#^	Actual Tm (°C)	C+G (%)	Length (bp)	Origin of primers
EL461013	HtEF1_F	TAACCGTTTCCGATCTGACC	51.1	55	52.6	19	This study
	HtEF1_R	TATGTCGCATCCACTCGAAG	51.1	55	40.9	22	
EL442868	HtGA2-ox_F	GGGTTCTTTAAGGTTGTTAATC	49.2	59	36.4	22	This study
	HtGA2-ox_R	TGATTTGCGGGTCTGTGTG	51.1	59	52.6	19	
EL469080	HtGA20-ox_F	AAGTAGCTTCACCGGGCG	52.6	55	61.1	18	This study
	HtGA20-ox_R	CTTGGTGTAGGATTGTCAAAGA	51.1	55	40.9	22	
EL458469	HtPHYB_F	GCTTCGTTGGTCAAGACGT	51.1	55	52.6	19	This study
	HtPHYB_R	CTTGTTGAACCCTTGTTTGATC	51.1	55	40.9	22	
SRAP primer	ME2	TGAGTCCAAACCGGAGC	49.5	50	58.8	17	Li and Quiros [29]
	ME5	TGAGTCCAAACCGGAAG	47.1	50	52.9	17	
	EM5	GACTGCGTACGAATTAAC	45.8	50	44.4	18	
	EM6	GACTGCGTACGAATTGCA	48.0	50	50.0	18	

^#^ Tm = annealing temperature.

## 4. Conclusions

We report an improved method MMY for high-quality and -quantity RNA extraction from dry seeds of Jerusalem artichoke with high levels of starch and lipid. The improved method is also applicable for seeds of sunflower, rice, maize and peanut, which are rich in polyphenols, lipids and polysaccharides. The effectiveness of the improved method to extract total RNA from seeds of these assayed plant species was confirmed with qPCR analysis of selected genes. 
